# Brain Metastases among Cancer Patients Diagnosed from 2010–2017 in Canada: Incidence Proportion at Diagnosis and Estimated Lifetime Incidence

**DOI:** 10.3390/curroncol29030169

**Published:** 2022-03-18

**Authors:** Jiaqi L. Liu, Emily V. Walker, Yuba Raj Paudel, Faith G. Davis, Yan Yuan

**Affiliations:** School of Public Health, University of Alberta, Edmonton, AB T6G 1C9, Canada; jiaqi8@ualberta.ca (J.L.L.); emily.walker@ualberta.ca (E.V.W.); yubaraj@ualberta.ca (Y.R.P.); faith.davis@ualberta.ca (F.G.D.)

**Keywords:** brain metastases, incidence proportion, lifetime incidence, lung cancer, Canada

## Abstract

The incidence of BM among Canadian cancer patients is unknown. We aimed to estimate IP of BM at the time of cancer diagnosis and during the lifetime of patients with selected primary cancers. Data on BM at diagnosis from 2010–2017 was obtained from the CCR. Site-specific IPs of BM were estimated from provincial registries containing ≥90% complete data on BM. The CCR IP estimates and the IP estimates from literature were applied to the total diagnosed primary cancers to estimate the number of concurrent BM and lifetime BM from 2010–2017 in Canada, respectively. The annual average number of patients with BM at diagnosis from all cancer sites was approximately 3227. The site-specific IPs of BM at diagnosis were: lung (9.42%; 95% CI: 9.16–9.68%), esophageal (1.58%; 95% CI: 1.15–2.02%), kidney/renal pelvis (1.33%; 95% CI: 1.12–1.54%), skin melanoma (0.73%; 95% CI: 0.61–0.84%), colorectal (0.22%; 95% CI: 0.18–0.26%), and breast (0.21%; 95% CI: 0.17–0.24%). Approximately 76,546 lifetime BM cases (or 5.70% of selected fifteen primary cancers sites) were estimated to have occurred from the 2010–2017 cancer patient cohort. These findings reflect results of population analyses in the US and Denmark. We recommend improved standardization of the collection of BM data within the CCR.

## 1. Introduction

Brain metastases from systemic malignancies (BM) occur almost twice as often as all primary malignant brain tumors including glioma [1,2]. Incidence rates of BM range from 7 to 14 persons per 100,000 population per year across population-based studies [3]. Incidence of BM is thought to be increasing mainly due to improved survival of patients owing to aggressive treatment of primary cancers [4], and improved detection of cancer due to wider introduction of advancing neuroimaging diagnostic technologies [5,6].

The primary cancers that most commonly metastasize to the brain are lung, breast, esophageal, skin melanoma, colorectal, and kidney/renal pelvis [5,6,7]. Among patients of these six cancers, 9% develop BM during their course of disease [5,8]. A population-based study from the US using cancer registry data found that over 27,000 (1.7%) were detected with BM at the time of diagnosis (from over 1.6 million primary cancers detected from 2010–2013) [9]. An earlier population-based study conducted in the US population estimated that 70,000 BM are expected to occur over the lifetime of 1,194,282 primary cancers patients diagnosed in the US in the year of 2007 [4]. Brain metastases at diagnosis may be a surrogate measure of cancer aggressiveness and/or diagnostic delay caused by patient or health care system related factors. In contrast, brain metastases lifetime occurrence in cancer patients is affected by more complex factors that include the above plus post-diagnosis system of care and survival factors. Population based estimates for BM in Canada are not available except for Alberta and Ontario [7,8].

In the last decade, treatments for brain metastases have started to improve what has been a dismal survival prognosis [10,11]. Survival rate of BM patients depends on the type and histology of the primary cancer, treatment of the primary cancer, and clinical characteristics such as age, size/number of BM, Karnofsky performance score, and effect of extra-cranial disease [11,12]. Treatment approaches for primary cancers and brain metastases (i.e., local therapy or systemic therapy) are crucial for the prognosis of patients with BM [5,10]. In addition to high rates of mortality, patients experience poor quality of life and various physical, cognitive, and neurological manifestations [13]. Hospitals and clinicians need to coordinate with brain cancer centers to achieve optimal outcomes for BM patients. 

The specific objectives of the current study are to estimate proportions of primary cancers (total, site, and histology) that metastasize to the brain using two time periods: at the time of diagnosis (hereafter referred to as concurrent BM) and over a cancer patients’ lifespan from the time of their diagnosis (hereafter referred to as lifetime BM). These data reflect the experience of Canadian patients with an initial diagnosis of primary cancer between 2010 and 2017. The lifetime numbers are estimates to help characterize the complete patient workload in the Canadian neuro-oncology community. The findings provide insight into the current and future needs of cancer patients at high risk for BM in Canada.

## 2. Materials and Methods

### 2.1. Data Source

In Canada, provincial and territorial cancer registries report cancer data to the Canadian Cancer Registry (CCR) managed by Statistics Canada. CCR includes population-based information on brain involvement at the time of diagnosis of a primary cancer, i.e., concurrent BM. We analyzed data collected on incidence of BM at diagnosis using the CCR 2017 version 2 data file 13. The data was accessed through the Statistics Canada’s Research Data Centre branch located at University of Alberta. Due to delays in reporting, undercounting of cases is most prominent in the last reported diagnosis year of 2017. The under-reporting is estimated to be 2% to 3% for all cancers combined and could contribute to the underestimation of both concurrent and lifetime BM counts [14].

### 2.2. Study Population

Canada has ten provinces and three territories with a population of 38.2 million in 2021. The 2017 CCR dataset has information on all Canadian residents (alive or dead) who had a new cancer diagnosis from 1992 to 2017. The CCR information on concurrent BM is available from most of the provinces and territories as the data in the CCR have been staged using the Collaborative Stage (CS) Data Collection System, which incorporates the TNM staging system. Distant metastatic involvement of the brain found at the time of diagnosis of primary cancer is coded within the CCR and metastases that occur after initial diagnosis are not recorded. Some provincial cancer registries code concurrent BM only for selected primary sites such as lung, breast, colorectal, and cervix. Logistical constraints limit the availability of data in the CCR from different provinces/territories to varying degrees. 

### 2.3. Inclusion/Exclusion Criteria

Cancer cases, regardless of age, diagnosed from 2010 to 2017 were included. Earlier data were not included in an effort to provide recent information and to be consistent with increasing focus on brain tumour data collection practices since 2010. To calculate concurrent BM incidence proportion (IP), we excluded cases from Quebec as data from Quebec is missing from the CCR. To utilize sparse data on some cancers, we grouped Eastern provinces (New Brunswick, Nova Scotia, Prince Edward Island, and Newfoundland and Labrador) into one regional group “Eastern provinces” and the Territories (Yukon, Nunavut, and Northwest territories) into another group “Territories”.

### 2.4. Cancer Selection and Classification

We investigated the proportion of concurrent BM in six primary cancer sites: lungs, breast, skin melanoma, colorectal, kidney/renal pelvis, and esophagus. These sites were chosen because previous findings from Alberta Cancer Registry data indicated that more than 90% of the brain metastases at diagnosis of primary cancers originated from these six sites [7]; a finding consistent with broader literature [9]. Remaining sites were categorized as “All other sites-combined”. Primary sites were coded using ICD-O-3 topographical/histological definitions and SEER site recodes (Table A1 in Appendix A). The most common cancers, lung and breast were further categorized. Lung cancers were grouped into non-small cell lung cancers (NSCLC) and small cell lungs cancers (SCLC) using ICD-O-3 histological codes. NSCLC histologies included adenocarcinoma, squamous cell carcinoma, and large cell lung carcinoma. Breast cancers were grouped into three categories representing hormone receptor status using combinations of estrogen receptors (ER), progesterone receptors (PR), and Human Epidermal Growth Factor Receptor 2 (HER2) results. These three groups are based on collaborative stage site-specific factor 16.

### 2.5. Data Analysis

All analysis was done with SAS 9.4. The current analyses involved two components: (1) estimation of concurrent BM using IP from CCR data and (2) population-based estimation of expected lifetime BM incidence IP from the literature applied to CCR data. Site specific IP was defined as the number of individuals diagnosed with brain metastases who had a specific single primary cancer divided by the total number of individuals diagnosed with that single primary cancer excluding individuals with missing data on BM. The IP was then expressed as a percentage. Crude IPs (IP_C_) (Table 1) were calculated from the data from all provinces and territories and was refined to minimize the effect of missing data (Table 2).

### 2.6. Estimation of Concurrent BM

To derive national estimates for concurrent BM incidence (for the selected six sites and “all other sites” combined), we calculated the total number of primary cancers and proportions of BM (number of BM at diagnosis divided by total number of new cancers) overall, by site and histology from the complete CCR datafile, excluding Quebec (Table 1). To minimize potential biases from regions with missing data, we calculated refined IP (IP_R_) estimates from only those provinces with >90% complete data in the BM at diagnosis variable as shown in Table 2. Within provinces with less than 10% missing data, the missing data was assumed to be missing completely at random. These IP_R_ BM estimates were then applied to the total number of patients of the selected primary cancer within the entire cohort to create national (Table 3) and provincial (Table A3) estimates. Quebec primary cancer data was only available in 2010. To impute Quebec total primary cancer cases, we applied the proportion of individuals with the specified primary cancer in 2010 to the midpoint Quebec population of each year between 2010–2017 and took the summation of the cases from every year.

### 2.7. Estimation of Expected Lifetime BM

Since we lacked data to directly estimate lifetime IP (IP_L_) of BM, we conducted a literature search for this information for our target primary sites (Table A2). The literature search was carried out using the MEDLINE database available from the University of Alberta library using the search terms ‘brain metastases’, ‘brain metastasis’, ‘incidence’ ‘incidence proportion’, combined with ‘[name of selected cancer sites]’. References within some papers were also reviewed to find additional studies. Studies identified through search and reference review were screened based on site of primary cancer and study objectives (measuring incidence of brain metastases). From the studies that passed through initial screening, following criteria were used to select the four most appropriate studies [5,8,15,16] for each selected primary cancer sites with lifetime IP estimates: (1) those with IP_L_ reflecting recent treatment approaches, (2) studies conducted in Canada or similar developed countries, (3) those with population based estimates, and (4) those with longer median follow up periods from diagnosis of primary cancers to ascertainment of BM status. Studies reporting IP_L_ based on autopsy findings were not considered.

To estimate the expected lifetime occurrence of BM, we applied site-specific IP_L_ obtained from the literature as described above (Table A2) to known national (Table 4) and provincial (Table A4) frequencies of primary cancers. National frequencies used for calculations of lifetime BM included the imputed Quebec population. We incorporated 15 primary sites into the lifetime estimates which accounts for 76.4% of the total new cases. The basis for exclusion for remaining 23.6% of total new cases include: in situ primary sites with smaller number of cases were not considered, unavailability of IP_L_ in the literature possibly due to limited incidence of BM, and primary malignant brain and central nervous system (CNS) tumours which don’t metastasize to the brain.

## 3. Results

There was a total of 1,396,390 primary cancers reported between 2010 and 2017 from nine provinces (excluding Quebec) (Table 1). Among them, 15,980 (1.14%) patients had concurrent BM. However, it was noted that among the six primary sites, breast and lung cancer had the least missing data at roughly 15%, colorectal had around 20% missing, while esophageal cancer, kidney/renal pelvis cancer, skin melanoma, and all other sites-combined had more than 60% missing data on concurrent BM (Table 1). Data completeness was largely dependent on reporting province (Table 2). Alberta reported no unknown/not applicable data on BM at diagnosis for all primary sites (Table 2).

### 3.1. Concurrent BM

Table 3 shows the estimated number of primary cancers diagnosed from 2010–2017, along with the estimated national counts for concurrent BM using IP_R_ from selected provinces with less than 10% missing BM data. We estimated that 3227 concurrent BM occurred annually in patients diagnosed between the 2010 to 2017 cohort (Table 3). This amounted to roughly 1.47% of the patients from all primary tumor sites developing concurrent brain metastases within the time period. The most frequent sites for concurrent BM per annum was lung (n = 2536) followed by skin melanoma (n = 99), kidney/renal pelvis (n = 83), breast (n = 65), colorectal (n = 59), and esophagus (n = 34). In addition, 352 concurrent BMs were estimated to occur annually from all other sites-combined per year.

Of total primary lung cancer cases from selected provinces, 9.42% (95% CI: 9.16–9.68%) presented with concurrent BM (Table 3). Among lung cancers, there was a higher IP_R_ among SCLC patients (13.21, 95% CI: 12.62–13.80) compared to NSCLC patients (9.55, 95% CI: 9.34–9.76). The next highest IP_R_ were observed in cancers at the following sites: esophagus (1.58%, 95% CI:1.15–2.02%), kidney/renal pelvis (1.33%, 95% CI: 1.12–1.54%), skin melanoma (0.73%, 95% CI: 0.61–0.84%), colon/rectum (0.22%, 95% CI: 0.18–0.26%), and breast (0.21%, 95% CI: 0.17–0.24%). Among breast cancer cases, triple negative hormone receptor status (ER^−^ PR^−^ HER2^−^) patients and HER2^+^ patients had higher IP_R_ (0.50%, 95% CI: 0.38–0.62 and 0.49%, 95% CI: 0.39–0.59, respectively) compared to those with ER^+^ PR^+^ HER2^−^ status (0.10%, 95% CI: 0.08–0.12). The IP_R_ for “all other sites-combined” was 0.31% (95% CI: 0.28–0.34), however as all provinces had greater than 10% missing data except for Alberta, this is essentially the Alberta IP. The concurrent development of BM across various cancer sites and provinces is shown in Table A3.

As Alberta data contained complete concurrent BM data for all primaries with no missing values, we opted to calculate the concurrent BM using IP from Alberta alone and compared them to selected provinces. Compared to selected provinces, Alberta has a higher IP for lung cancer (10.13% in Alberta vs. 9.42%), while it has a lower IP for breast cancer (0.15% vs. 0.21%). The IPs for skin melanoma, kidney/renal pelvis, and colorectal were similar or slightly lower for Alberta compared to the selected province sample. Using the Alberta IP to estimate the annual concurrent BM within the 2010–2017 Canadian cohort, an additional 191 concurrent BM (annual estimate from Alberta IP: 2727) for patients with lung cancer and an additional 149 concurrent BM (annual estimate from Alberta IP: 3376) for all primaries were estimated to occur. Most excess BM cases from Alberta were contributed from lung cancer diagnoses. Alberta had a similar survival rate among lung cancer patients compared to other Canadian provinces but a higher proportion of lung cancer diagnosis at later stage (Stage III and IV) than other provinces except Saskatchewan, Prince Edward Island, and Territories [17]. As such, using IP from Alberta alone to create national estimates may misrepresent the Canadian cancer patient population. This sub-analysis further indicates that the estimates using multiple selected provinces roughly remains similar to the estimates using only Alberta where there is no missing data. As such, we elected to report estimates in Table 3 using the IP_R_ from multiple provinces as described above.

### 3.2. Lifetime BM

Between 2010 and 2017, an estimated 1,759,360 new cancer cases were diagnosed in Canada. Among them, 1,343,620 (76%) were from selected 15 sites for which IP_L_ estimates were available in the literature. Applying the incidence proportions from literature (Table A2), 76,546 cases of lifetime BM are estimated to develop within the Canadian 2010–2017 cohort (Table 4). The estimated distribution of lifetime BM is 42,895 from lung cancer; 12,524 from breast cancers; 7449 from skin melanomas, 4013 from colorectal cancers, 3239 from kidney cancers, 887 from esophageal cancers, and 5646 from the other nine primary cancer sites (Table 4). Among the six cancer sites described above, 8.32% of patients are expected to develop cancer over the course of their disease (Table 4), comparable to that of the US [5,8]. The lifetime development of BM across various cancer sites and provinces is shown on Table A4.

## 4. Discussion

This study was undertaken to estimate the expected numbers of brain metastases in Canada. It is estimated that nearly 3227 concurrent BM occur with all cancer sites each year. The IP_R_ for the six cancer sites (lung, breast, skin melanoma, colorectal, kidney/renal pelvis, and esophagus) were similar to findings reported in studies conducted in the US and the Netherlands [9,18]. The highest IP_R_ and frequency of concurrent BM (9.42% and 2536 annually) was observed among lung cancer cases. Cancer cells from most cancer primaries must achieve passage through the lungs to reach the brain. However, there is direct circulation of lung cancer cells from the pulmonary venous system, through the heart to the brain which makes lung cancer the most commonly metastasizing cancer to the brain [9]. Furthermore, almost half of the lung cancer in Canada between 2011–2017 were stage IV, i.e., diagnosed at stage IV after they have metastasized [19]. The incidence proportion of BM was higher among SCLC (13.21%) patients compared to NSCLC (9.55%) patients in the current study. Similar findings were reported in a US study where the proportion of BM at diagnosis was 15.1% and 10.7%, respectively [9]. More than two-thirds of SCLC (67.4%) were diagnosed at stage IV in Canada between 2011–2015 compared to below half of the NSCLCs (47.1%) [20]. The later diagnosis stages of SCLC may play a confounding role in the increase in IP of BM in comparison to NSCLC which has a greater percentage of cases diagnosed at an earlier stage.

The IP_R_ of BM in breast cancer patients in the current analysis was 0.21%, while in the US the incidence proportion was 0.41% from the 2010–2013 period [21]. Comparing primary sites that have similar IP_L_, the IP_R_ for breast cancer is relatively low at 0.21% (IP_L_: 5.08%) in comparison to esophagus at 1.58% (IP_L_: 5.19%) and kidney/renal pelvis at 1.33% (IP_L_: 6.48%). Although incidence of BM from breast cancer is thought to be increasing in recent years [22,23], metastatic breast tumours migrate to the brain later in the course of disease than to the liver, lung, or bone [24]. Additionally, organized breast cancer screening programs in Canada might have led to detection of breast cancers at a localized stage when brain metastases are less likely to occur, as less than 5% of breast cancers were diagnosed at stage IV between 2011–2015 in Canada [20]. Among breast cancer patients, triple negative hormone receptor status (ER^−^ PR^−^ HER2^−^) and HER2^+^ showed a higher risk of developing BM at the time of diagnosis compared to ER^+^ PR^+^ HER2^−^ breast cancers. Triple negative breast cancers comprise 10–15% of all breast cancers and have a higher incidence and earlier development of BM in comparison to other hormone receptor status [25,26]. Analysis of SEER data among patients who received a breast cancer diagnosis from 2010 to 2014 showed that HER2^+^ (1%) and triple negative breast cancer (0.7%) had higher incidence of BM than other subtypes [27]. HER2^+^ and triple negative breast cancers had the highest rate of metastases in lung and liver compared to other subtypes. It is suggested that HER2 over-expression may be associated with increased propensity to migrate to the lungs or brain [28].

The number of BM occurring from skin melanoma is the third highest in comparison to most other primary sites with a relatively high IP_L_ of 6.88%. The higher propensity of melanoma to migrate to the brain—higher than any other primary site for tumours diagnosed at later stages—was also reported using the metropolitan Detroit Cancer surveillance system data [5]. A Canadian Cancer Society report showed that 14.3% of melanoma diagnosed between 2011–2015 were diagnosed at stage III or stage IV [17]. A study from Ontario, Canada found that among patients with metastatic melanoma who were tested for BRAF mutation, more than one-third (38.3%) tested positive [29]. The BRAF mutation causes constitutive activation of the MAPK/ERK pathways promoting rampant cell growth and division, and it has been suggested that inhibition of the B-Raf protein may be an important strategy in inhibiting advanced stages of metastatic melanoma disease [30]. Targeted therapies often work against metastatic lesions for a time among melanoma patients, however, BM eventually develop resistance to targeted therapies [31,32]. Melanoma cells are suggested to adopt a phenotypic switching mechanism to resist the effect of drugs and gain a greater ability to metastasize under targeted therapy [31]. Among targeted therapies used, immunotherapy administration of anti-CTLA-4 antibodies has been described as leading to the uncommon occurrence of pseudoprogression of melanoma, potentially causing new lesions [33]. However, subsequent tumor repression is observed with this phenomenon. This may lead to false positives of BM diagnoses leading to overestimation of BM incidence cases with the increasing prevalence of immunotherapy usage.

This analysis showed that there are a high proportion of unknown status of BM at the time of diagnosis with greater than 15% missing for the six cancer sites: lung, breast, skin melanoma, colorectal, kidney/renal pelvis, and esophagus. Accurate coding and staging of cancer cases is a time-consuming process that requires highly trained cancer registrars with subject matter expertise in staging systems and cancer-specific factors. The varying degrees of missingness across province/territory likely reflects resource constraints, which limit person-time available to complete staging for all cancers. High quality data on metastases, and more broadly stage at diagnosis, is necessary for efficient health system planning, and monitoring the impacts of health system changes and service interruptions. This analysis highlights that there is an unacceptable amount of missing data across Canada and underscores the need to advocate for resources to support provincial/territorial cancer registries in capturing more complete data. The reporting of the Alberta Cancer Registry provides a snapshot of the distribution of BMs over all primaries that can be expected if all primary sites had BM documented. 

Among the cancer patients with initial diagnosis of cancer in 15 primary cancer sites that commonly metastasize to the brain, 76,546 lifetime BM case (concurrent BM and those diagnosed during follow up) were estimated to occur within the 2010–2017 Canadian cohort equating to 5.70% of cohort. This is slightly over three times the number of primary malignant brain tumors diagnosed from 2010–2017 in Canada [34]. For estimating lifetime BM incidence, we included 76.4% of the newly diagnosed primary cancers between 2010 and 2017. As the remaining 23.6% of total new cases were not considered for this report due to previously mentioned reasons, the total lifetime BM cases within the total 2010–2017 Canadian cohort could not be estimated. The 76,546 lifetime BM cases calculated from the 15 sites are an underestimation of the total lifetime BM cases within the Canadian cohort.

The lack of consistent recording of brain metastases for all primary cancers across provinces limits the accuracy of estimates for concurrent incidence of site specific and overall BM. The potential under coverage for rare cancers (skin melanoma, esophagus, kidney, and others) would also result in an overall BM underestimation [35]. As we applied the same IP to all provinces of Canada (from literature for lifetime BM and from the CCR for concurrent BM) we may also be missing diversity among regions. The IP_R_ of BM at diagnosis from the selected province sample for lung, breast, skin melanoma, and colorectal cancer was lower than in the IP_C_ estimate from the total sample. This might have caused underestimation of national and provincial estimates for BM of these cancer sites. The lower IP_R_ in our selected sample may be due to higher IPs recorded in the excluded provinces creating a recording bias if recording routinely occurred when BM were present and not routinely recorded if this information was unknown or not present. 

Accuracy of our lifetime estimates depends upon quality of data from selected studies. Since the paper describing IP_L_ in Ontario by Habbous et. al. included a limited number of years, 2010 up to 2018, the estimation of the IP_L_ we used might be lower than the underlying actual value [8]. This underestimation would lead to lower lifetime estimates. Additionally, since Quebec data after 2010 was not available, estimates for Quebec were based only on 2010 data while those for other provinces were based on the average of 8 years of data (2010–2017). Furthermore, as lifetime estimates of brain metastases only included 15 primary sites, there were primary cancer cases outside of the 15 primary sites analyzed (excluding CNS tumors) that were not included. The lifetime brain metastases of the total Canadian cohort from 2010–2017 could not be calculated due to this lack of information from the exclusion criteria as described above.

To our knowledge, national level estimates for concurrent or lifetime BM for primary cancer types have not been previously published in Canada. Our report gives important information about a group of patients that have a significant need for supportive or palliative care which may incur high health-care costs. More research is needed to understand risk factors for BM, examine factors influencing survival of patients with BM, and understand the changing distributions of BM by patient and disease characteristics. As such, provincial cancer registries need support to improve the standardization of BM data collection. Estimating frequency of BM by population sub-groups will facilitate specialty care centers to plan patient services. With changes in patterns of primary cancer incidence, population dynamics, emerging treatment methods and access to care, population level BM incidence in Canada needs to be regularly monitored.

## 5. Conclusions

Using Canadian Cancer registry data and recent clinical experience from the literature, we present the first national estimates of BM incidence. The proportion (IP) of concurrent metastases to the brain were: lung cancer (9.42%), esophageal (1.58%), kidney/renal pelvis (1.33%), skin melanoma (0.73%), colorectal (0.22%), and breast (0.21%) cancers. Both the refined IP and actual numbers of patients with metastases to the brain at diagnosis were highest for lung cancer (n = 2536). It is estimated that over 3227 individuals with concurrent brain metastases among new cancer diagnoses present annually. Using the available clinical and population data, we estimate that approximately 76,546 BM patients will be diagnosed in the 2010–2017 Canadian cancer cohort over their lifetime. This is an underestimate as were limited to the 15 primary sites with lifetime estimates for these secondary tumours. 

We previously estimated that approximately 2500 individuals with malignant brain tumours are newly diagnosed each year in Canada [34], with a total of 20,000 malignant brain tumour diagnosis between 2010 to 2017. These current findings indicate that another 76,546 individuals with other types of cancer will present with subsequent brain metastases creating approximately 96,546 malignant brain tumour patients within the lifetimes of the 2010–2017 cohort for neuro-oncology specialists to address. Future estimates will include those patients with an initial non-malignant brain tumour diagnosis. With changes in patterns of primary cancer incidence, population dynamics, emerging treatment methods, and access to care, population level BM incidence in Canada needs to be regularly monitored. With the continual emergence of new data on brain metastases, we encourage the CCR to work towards standardizing the way these data are collected.

## Figures and Tables

**Table 1:** Frequency of concurrent brain metastases and crude incidence proportions (IPc) in Canada by selected cancer sites (2010–2017).

Cancer Site	Sub-Category/Histology	Number of Primary Cases *	IP_c_ (%)	Brain Metastasis at Diagnosis					
				Missing	%	No	%	Yes	%
Lung	All	160,525	10.40	24,805	15.45	121,600	75.75	14,120	8.80
	NSCLC	104,770	9.37	11,630	11.10	84,080	80.25	9060	8.65
	SCLC	16,620	13.39	1725	10.38	12,900	77.62	1995	12.00
Breast	All	193,970	0.24	29,485	15.20	164,085	84.59	400	0.21
	ER^−^ PR^−^ HER2^−^	12,945	0.50	70	0.54	12,810	98.96	65	0.50
	HER2^+^	19,325	0.49	115	0.60	19,115	98.91	95	0.49
	ER^+^ PR^+^ HER2^−^	86,185	0.10	570	0.66	85,530	99.24	85	0.10
Skin melanoma		98,080	0.28	69,145	70.50	28,665	29.23	270	0.28
Colorectal		168,610	0.20	34,120	20.24	134,150	79.56	340	0.20
Kidney/Renal Pelvis		39,590	0.39	27,905	70.48	11,530	29.12	155	0.39
Esophagus		14,145	0.39	10,665	75.40	3425	24.21	55	0.39
All other sites		721,470	0.09	453,760	62.89	267,070	37.02	640	0.09
Total		1,396,390	2.14	649,885	46.54	730,525	52.32	15,980	1.14

* Excluding Quebec.

**Table 2:** Percentage of ‘unknown and not applicable data’ on brain metastases at diagnosis in Canada by province/territories by primary cancer sites (2010–2017).

Province/Territories	Lung (%)	Breast (%)	Skin Melanoma (%)	Colorectal (%)	Kidney/Renal Pelvis (%)	Esophagus (%)	All other Sites-Combined (%)
Alberta	**0.00**	**0.00**	**0.00**	**0.00**	**0.00**	**0.00**	**0.00**
British Columbia	15.82	**3.06**	100.00	25.62	100.00	100.00	80.76
Manitoba	**2.54**	**1.19**	**8.84**	**2.38**	**4.91**	**1.85**	26.33
New Brunswick	**6.02**	**1.18**	100.00	**5.77**	100.00	100.00	75.23
Newfoundland and Labrador	**2.99**	**1.23**	10.15	17.21	**2.59**	18.18	23.03
Nova Scotia	**2.47**	**2.04**	**4.00**	**3.89**	**2.18**	**2.24**	25.38
Ontario	15.41	21.08	86.88	23.71	100.00	100.00	78.34
Prince Edward Island	**7.34**	**3.07**	**3.68**	**4.63**	**4.17**	15.00	23.97
Quebec	100.00	100.00	100.00	100.00	100.00	100.00	100.00
Saskatchewan	**4.22**	**2.04**	**7.89**	**3.03**	**2.68**	**3.37**	25.66
Territories	10.42	**8.42**	55.56	11.76	45.45	66.67	50.35

Provinces with less than 10% missing data on concurrent brain metastases diagnosis were bolded and their data were used to calculate IP_R_ in Table 3.

**Table 3:** Estimated refined concurrent incidence proportion (IP_R_) and frequency of BM in Canada by selected cancer sites adjusting for missing data (2010–2017).

Cancer Site	Sub-Category/Histology	Total Number of Primary Cancer Cases **	IP_R_ from Selected Provinces (95%CI)	Estimated Concurrent BM Incidence Cases from IP_R_	Average Annual Estimates of Concurrent BM
Lung	All	215,335	9.42 (9.16–9.68) ^1^	20,285	2536
	NSCLC	136,725	9.55 (9.34–9.76) ^2^	13,057	1632
	SCLC	22,270	13.21 (12.62–13.80) ^3^	2921	365
Breast	All	246,545	0.21 (0.17–0.24) ^4^	518	65
	ER^−^ PR^−^ HER2^−^ *	12,945	0.50 (0.38–0.62) ^5^	65	8
	HER2^+^ *	19,325	0.49 (0.39–0.59) ^5^	95	12
	ER^+^ PR^+^ HER2^−^ *	86,185	0.10 (0.08–0.12) ^5^	86	11
Skin melanoma		108,265	0.73 (0.61–0.84) ^6^	790	99
Colorectal		214,615	0.22 (0.18–0.26) ^7^	472	59
Kidney/Renal Pelvis		49,984	1.33 (1.12–1.54) ^8^	665	83
Esophagus		17,110	1.58 (1.15–2.02) ^9^	270	34
All other sites-combined		907,505	0.31 (0.28–0.34) ^10^	2813	352
Total (from all sites)		1,759,360	1.47	25,813	3227

Notes (selected provinces/territories): ^1^ Alberta, Manitoba, New Brunswick, Newfoundland and Labrador, Nova Scotia, Prince Edward Island, Saskatchewan; ^2^ Alberta, Manitoba, New Brunswick, Newfoundland and Labrador, Nova Scotia, Ontario, Prince Edward Island, Saskatchewan, Territories; ^3^ Alberta, Manitoba, New Brunswick, Newfoundland and Labrador, Nova Scotia, Ontario, Prince Edward Island, Saskatchewan; ^4^ Alberta, Manitoba, New Brunswick, Newfoundland and Labrador, Nova Scotia, Prince Edward Island, Saskatchewan, Territories; ^5^ Alberta, British Columbia, Manitoba, New Brunswick, Newfoundland and Labrador, Nova Scotia, Ontario, Prince Edward Island, Saskatchewan, Territories; ^6^ Alberta, Manitoba, Nova Scotia, Prince Edward Island, Saskatchewan; ^7^ Alberta, Manitoba, New Brunswick, Nova Scotia, Prince Edward Island, Saskatchewan; ^8^ Alberta, Manitoba, Newfoundland and Labrador, Nova Scotia, Prince Edward Island, Saskatchewan; ^9^ Alberta, Manitoba, Nova Scotia, Saskatchewan; ^10^ Only Alberta. * Hormone receptor status not available for breast cancer in Quebec data from 2010; ** Due to unavailability of data, incidence of cancer for 2011–2017 is assumed to be the same as 2010 for Quebec adjusted for population growth.

**Table 4:** Estimated lifetime incidence proportion (IP_L_) and frequency of brain metastases by selected primary site (2010–2017) for all Canadian provinces and territories.

Cancer Site	Sub-Category/Histology	Total Number of Primary Cancer Cases **	IP_L_ (Table A2)	Estimated Lifetime BM Incidence Cases From IP_L_
Lung	All	215,335	19.92	42,895
	NSCLC	136,725	8.91	12,262
	SCLC	22,270	17.95	3997
Breast	All	246,545	5.08	12,524
	ER^−^ PR^−^ HER2^−^ *	12,945	5.85	757
	HER2^+^ *	19,325	5.63	1088
	ER^+^ PR^+^ HER2^−^ *	86,185	1.54	1327
Skin melanoma		108,265	6.88	7449
Colorectal		214,615	1.82	3906
Kidney		49,984	6.48	3239
Esophagus		17,110	5.19	887
Stomach		31,535	2.14	675
Liver		18,070	1.02	184
Uterus		49,025	1.30	637
Ovary		26,250	2.06	541
Prostate		172,990	0.66	1142
Testes		8930	1.30	116
Urinary bladder		84,390	1.35	1139
Thyroid gland		47,795	0.48	229
Non-Hodgkin lymphoma		52,790	1.86	982
Total of 15 sites		1,343,620	5.70	76,546

* Hormone receptor status not available for breast cancer in Quebec data from 2010. ** Due to unavailability of data, incidence of cancer for 2011–2017 was assumed to be same as 2010 for Quebec adjusted for population growth.

## Data Availability

Third Party Data: Restrictions apply to the availability of these data. Data was obtained from the Canadian Cancer Registry and are available from the Research Data Center program with the permission of Statistics Canada.

## Appendix A

**Table A1:** Cancer Definitions by SEER ICD-O-3 codes.

Cancer	ICD-O-3(Site/Type)
Lung	C340-C343, C348–349
Breast	C500-C506, C508–509
Skin melanoma	C440-C449
Colorectal	C180-C189, C199, C209
Kidney/Renal pelvis	C649, C659
Esophagus	C150-C155, C158, C159
Stomach	C160-C166, C168-C169
Liver	C220
Uterus	C540-C543, C548-C549, C559
Ovary	C569
Prostate	C619
Testes	C620-C621, C629
Urinary bladder	C670-C679
Thyroid	C739
Non-Hodgkin lymphoma	9590–9597, 9670–9719, 9724–9729, 9735, 9737, 9738 9811–9818, 9823, 9827, 9837 all sites except C42.0,.1,.4
Lung: NSCLC	Adenocarcinoma: 8140, 8211, 8230– 8231, 8250–8260, 8323, 8480–8490, 8550–8551, 8570–8574, 8576 Squamous cell carcinoma: 8050–8078 and 8083–8084 Large cell lung carcinoma: 8010–8012, 8014–8031, 8035, 8046, 8310.
Lung: SCLC	8041–8045
Breast: ER^−^, PR^−^, HER2^−^	Collaborative stage site-specific factor 16: 000
Breast: HER2^+^	Collaborative stage site-specific factor 16: 001, 101,011, and 111
Breast: ER^+^, PR^+^, HER2^−^	Collaborative stage site-specific factor 16: 110.

**Table A2:** Lifetime incidence proportion (IP_L_) of BM from selected studies by site of cancer.

Authors, Year	Country/Region	Study Time Period	Study Population	Primary Site	Median (IQR) Duration to BM Onset (Months)	IP_L_ (%)
Barnholtz-Sloan et al., 2004 [5]	Detroit, Michigan, USA	1973–2001	Patients diagnosed with primary cancer, population-based study with chart review	Lung	ns	19.92
				Breast	ns	5.08
				Melanoma	ns	6.88
				Colorectal	ns	1.82
				Kidney	ns	6.48
Habbous et al., 2020 [8]	Ontario, Canada	2010–2019	Adult Ontario residents with valid health card number diagnosed with primary cancer within the Ontario Cancer Registry	Esophagus	5.9 (1.1, 13.7)	5.19
				Liver	12.8 (1.1, 29.4)	1.02
				Non-Hodgkin lymphoma	Nodal: 7.8 (3.5, 13.6) Extranodal: 6.7 (2.2, 13.6)	1.86
				Ovary	20.5 (13.0, 40.5)	2.06
				Prostate	21.6 (7.2, 44.8)	0.66
				Testes	5.6 (1.5, 9.0)	1.30
				Thyroid	18.1 (5.3, 40.6)	0.48
				Urinary Bladder	11.0 (4.6, 20.2)	1.35
				Uterus	14.9 (6.5, 32.3)	1.30
				Stomach	8.8 (1.3, 19.0)	2.14
Gonclaves et al., 2016 [15]	Detroit, Michigan, USA	1973–2011	Patients diagnosed with nonmetastatic first primary lung cancer in the Metropolitan Detroit Surveillance, Epidemiology, and End Results (SEER) registry	Lung, SCLC	ns	17.94
				Lung, NSCLC	ns	8.91
Arvold et al., 2012 [16]	Boston, Massachusetts, USA	1997–2006	Women with clinical stage I or II invasive breast cancer who received breast conserving therapy	Breast, ER^−^ PR^−^ HER2^−^	37.1 (8.9, 58.7)	5.85
				Breast, HER2^+^	Luminal-HER2: 55.0 (24.3–102.8) HER2: 34.5 (18.9–107.6)	5.63
				Breast, ER^+^ PR^+^ HER2^−^	Luminal A: 65.7 (27.4–76.7) Luminal B: 63.7 (7.6–106.1)	1.54

ns: not specified.

**Table A3:** Average annual estimates of concurrent brain metastases by provinces/territories among cancer patients diagnosed in Canada (2010–2017) by selected primary cancer sites.

Number of Cases	Cancer Sites	Region/Province								
		Eastern Provinces	Quebec *	Ontario	Manitoba	Saskatchewan	Alberta	British Columbia	Territories	Total
Concurrent BM	Lung	225	735	907	86	74	202	302	6	2536
	Breast	5	16	26	2	2	6	8	0	65
	Skin melanoma	9	11	52	3	2	9	13	0	99
	Colorectal	6	14	22	3	2	5	7	0	59
	Kidney/Renal Pelvis	8	20	31	3	3	8	10	0	83
	Esophagus	3	7	14	1	1	3	5	0	34
	All other sites	25	82	146	12	10	33	44	1	352
	Total	280	884	1198	110	93	265	390	7	3227
Total Primary Cancers	Lung	2384	7803	9625	911	784	2141	3209	60	26,917
	Breast	2258	7485	12,222	994	858	2901	4041	59	30,818
	Skin melanoma	1191	1450	7106	459	285	1189	1842	11	13,533
	Colorectal	2503	6548	9898	1208	969	2326	3311	64	26,825
	Kidney/Renal Pelvis	629	1481	2358	254	210	584	718	14	6249
	Esophagus	178	420	907	68	56	193	311	6	2137
	All other sites	8136	26,483	47,078	3865	3069	10,520	14,109	178	113,435
	Total	17,278	51,671	89,192	7760	6230	19,853	27,540	392	219,915

* Due to unavailability of data, annual average estimates for Quebec used 2010 frequencies adjusted for population growth.

**Table A4:** Average number of lifetime brain metastases by provinces/territories among cancer patients diagnosed in Canada (2010–2017) by primary cancer sites.

Number of Cases	Cancer Sites	Region/Province								
		Eastern Provinces	Quebec *	Ontario	Manitoba	Saskatchewan	Alberta	British Columbia	Territories	Total
Lifetime BM	Lung	3799	12,435	15,338	1451	1250	3412	5113	96	42,895
	Breast	918	3042	4967	404	349	1179	1642	24	12,524
	Skin melanoma	656	798	3911	253	157	654	1014	6	7449
	Colorectal	364	954	1441	176	141	339	482	9	3906
	Kidney	326	768	1222	132	109	303	372	7	3239
	Esophagus	74	174	377	28	23	80	129	2	887
	Stomach	53	150	300	24	20	51	77	1	675
	Liver	10	36	78	5	4	18	33	0	184
	Uterus	48	136	267	27	18	60	80	1	637
	Ovary	39	144	200	20	17	46	74	1	541
	Prostate	96	238	451	37	36	122	160	2	1142
	Testes	7	23	47	4	4	15	16	0	116
	Urinary bladder	88	304	435	35	33	96	148	1	1139
	Thyroid gland	15	50	119	6	4	19	17	0	229
	Non-Hodgkin lymphoma	75	290	346	35	26	86	122	2	982
	Total of 15 sites	6567	19,543	29,499	2636	2190	6478	9479	154	76,546
Total Primary Cancers	Lung	19,070	62,425	77,000	7285	6275	17,130	25,670	480	215,335
	Breast	18,065	59,880	97,775	7955	6865	23,205	32,325	475	246,545
	Skin melanoma	9530	11,600	56,845	3675	2280	9510	14,735	90	108,265
	Colorectal	20,020	52,395	79,180	9665	7755	18,605	26,485	510	214,615
	Kidney	5035	11,845	18,865	2035	1680	4675	5745	110	49,990
	Esophagus	1425	3360	7255	540	445	1540	2485	45	17,095
	Stomach	2460	7010	14,020	1105	930	2365	3580	65	31,535
	Liver	985	3525	7680	500	375	1745	3220	40	18,070
	Uterus	3715	10,495	20,530	2095	1370	4600	6145	75	49,025
	Ovary	1905	7010	9695	985	815	2215	3585	40	26,250
	Prostate	14,515	36,110	68,400	5550	5445	18,415	24,295	260	172,990
	Testes	520	1805	3595	340	280	1150	1205	35	8930
	Urinary bladder	6495	22,500	32,200	2590	2455	7095	10,955	100	84,390
	Thyroid gland	3090	10,410	24,750	1180	820	4010	3480	55	47,795
	Non-Hodgkin lymphoma	4055	15,575	18,620	1860	1415	4615	6565	85	52,790
	Total of 15 sites	110,885	315,945	536,410	47,360	39,205	120,875	170,475	2465	1,343,620

* Due to unavailability of data, annual average estimates Quebec used 2010 frequencies adjusted for population growth.

## References

[1] Chamberlain M.C., Baik C.S., Gadi V.K., Bhatia S., Chow L.Q.M. (2017). Systemic Therapy of Brain Metastases: Non–Small Cell Lung Cancer, Breast Cancer, and Melanoma. Neuro-Oncology.

[2] Saria M., Piccioni D., Carter J., Orosco H., Turpin T., Kesari S. (2015). Current Perspectives in the Management of Brain Metastases. Clin. J. Oncol. Nurs..

[3] Fox B.D., Cheung V.J., Patel A.J., Suki D., Rao G. (2011). Epidemiology of Metastatic Brain Tumors. Neurosurg. Clin. North Am..

[4] Davis F.G., Dolecek T.A., McCarthy B.J., Villano J.L. (2012). Toward Determining the Lifetime Occurrence of Metastatic Brain Tumors Estimated from 2007 United States Cancer Incidence Data. Neuro-Oncology.

[5] Barnholtz-Sloan J.S., Sloan A.E., Davis F.G., Vigneau F.D., Lai P., Sawaya R.E. (2004). Incidence Proportions of Brain Metastases in Patients Diagnosed (1973 to 2001) in the Metropolitan Detroit Cancer Surveillance System. J. Clin. Oncol..

[6] Smedby K.E., Brandt L., Bäcklund M.L., Blomqvist P. (2009). Brain Metastases Admissions in Sweden between 1987 and 2006. Br. J. Cancer.

[7] Villano J.L., Durbin E.B., Normandeau C., Thakkar J.P., Moirangthem V., Davis F.G. (2015). Incidence of Brain Metastasis at Initial Presentation of Lung Cancer. Neuro-oncology.

[8] Habbous S., Forster K., Darling G., Jerzak K., Holloway C.M.B., Sahgal A., Das S. (2021). Incidence and Real-World Burden of Brain Metastases from Solid Tumors and Hematologic Malignancies in Ontario: A Population-Based Study. Neuro-Oncol. Adv..

[9] Kromer C., Xu J., Ostrom Q.T., Gittleman H., Kruchko C., Sawaya R., Barnholtz-Sloan J.S. (2017). Estimating the Annual Frequency of Synchronous Brain Metastasis in the United States 2010–2013: A Population-Based Study. J. Neurooncol..

[10] Berghoff A.S., Schur S., Füreder L.M., Gatterbauer B., Dieckmann K., Widhalm G., Hainfellner J., Zielinski C.C., Birner P., Bartsch R. (2016). Descriptive Statistical Analysis of a Real Life Cohort of 2419 Patients with Brain Metastases of Solid Cancers. ESMO Open.

[11] Soffietti R., Abacioglu U., Baumert B., Combs S.E., Kinhult S., Kros J.M., Marosi C., Metellus P., Radbruch A., Villa Freixa S.S. (2017). Diagnosis and Treatment of Brain Metastases from Solid Tumors: Guidelines from the European Association of Neuro-Oncology (EANO). Neuro-Oncology.

[12] Klos K.J., O’Neill B.P. (2004). Brain Metastases. Neurologist.

[13] Lowery F.J., Yu D. (2017). Brain Metastasis: Unique Challenges and Open Opportunities. Biochim. Biophys. Acta (BBA)—Rev. Cancer.

[14] Government of Canada S.C. Canadian Cancer Registry (CCR). https://www23.statcan.gc.ca/imdb/p2SV.pl?Function=getSurvey&SDDS=3207.

[15] Goncalves P.H., Peterson S.L., Vigneau F.D., Shore R.D., Quarshie W.O., Islam K., Schwartz A.G., Wozniak A.J., Gadgeel S.M. (2016). Risk of Brain Metastases in Patients with Nonmetastatic Lung Cancer: Analysis of the Metropolitan Detroit Surveillance, Epidemiology, and End Results (SEER) Data. Cancer.

[16] Arvold N.D., Oh K.S., Niemierko A., Taghian A.G., Lin N.U., Abi-Raad R.F., Sreedhara M., Harris J.R., Alexander B.M. (2012). Brain Metastases after Breast-Conserving Therapy and Systemic Therapy: Incidence and Characteristics by Biologic Subtype. Breast Cancer Res. Treat..

[17] (2018). Canadian Cancer Statistics 2018; Canadian Cancer Statistics Advisory Committee in collaboration with the Canadian Cancer Society, Statistics Canada and the Public Health Agency of Canada. https://www150.statcan.gc.ca/n1/daily-quotidien/180612/dq180612e-eng.htm.

[18] Schouten L.J., Rutten J., Huveneers H.A.M., Twijnstra A. (2002). Incidence of Brain Metastases in a Cohort of Patients with Carcinoma of the Breast, Colon, Kidney, and Lung and Melanoma. Cancer.

[19] (2021). Canadian Cancer Statistics 2021; Canadian Cancer Statistics Advisory Committee in collaboration with the Canadian Cancer Society, Statistics Canada and the Public Health Agency of Canada. https://www.canada.ca/en/public-health/services/reports-publications/health-promotion-chronic-disease-prevention-canada-research-policy-practice/vol-41-no-11-2021/canadian-cancer-statistics-2021.html.

[20] Bryan S., Masoud H., Weir H.K., Woods R., Lockwood G., Smith L., Brierley J., Gospodarowicz M., Badets N. (2018). Cancer in Canada: Stage at Diagnosis. Health Rep..

[21] Cagney D.N., Martin A.M., Catalano P.J., Redig A.J., Lin N.U., Lee E.Q., Wen P.Y., Dunn I.F., Bi W.L., Weiss S.E. (2017). Incidence and Prognosis of Patients with Brain Metastases at Diagnosis of Systemic Malignancy: A Population-Based Study. Neuro-Oncology.

[22] Tabouret E., Chinot O., Metellus P., Tallet A., Viens P., Gonçalves A. (2012). Recent Trends in Epidemiology of Brain Metastases: An Overview. Anticancer Res..

[23] Frisk G., Svensson T., Bäcklund L.M., Lidbrink E., Blomqvist P., Smedby K.E. (2012). Incidence and Time Trends of Brain Metastases Admissions among Breast Cancer Patients in Sweden. Br. J. Cancer.

[24] Lin N.U., Bellon J.R., Winer E.P. (2004). CNS Metastases in Breast Cancer. J. Clin. Oncol..

[25] Mittendorf E.A., Philips A.V., Meric-Bernstam F., Qiao N., Wu Y., Harrington S., Su X., Wang Y., Gonzalez-Angulo A.M., Akcakanat A. (2014). PD-L1 Expression in Triple-Negative Breast Cancer. Cancer Immunol. Res..

[26] Berghoff A.S., Preusser M. (2018). New Developments in Brain Metastases. Ther. Adv. Neurol. Disord.

[27] Kim Y.-J., Kim J.-S., Kim I.A. (2018). Molecular Subtype Predicts Incidence and Prognosis of Brain Metastasis from Breast Cancer in SEER Database. J. Cancer Res. Clin. Oncol..

[28] Hicks D.G., Short S.M., Prescott N.L., Tarr S.M., Coleman K.A., Yoder B.J., Crowe J.P., Choueiri T.K., Dawson A.E., Budd G.T. (2006). Breast Cancers with Brain Metastases Are More Likely to Be Estrogen Receptor Negative, Express the Basal Cytokeratin CK5/6, and Overexpress HER2 or EGFR. Am. J. Surg. Pathol..

[29] Ernst D.S., Petrella T., Joshua A.M., Hamou A., Thabane M., Vantyghem S., Gwadry-Sridhar F. (2016). Burden of Illness for Metastatic Melanoma in Canada, 2011-2013. Curr. Oncol..

[30] Davies H., Bignell G.R., Cox C., Stephens P., Edkins S., Clegg S., Teague J., Woffendin H., Garnett M.J., Bottomley W. (2002). Mutations of the BRAF Gene in Human Cancer. Nature.

[31] Haueis S.A., Kränzlin P., Mangana J., Cheng P.F., Urosevic-Maiwald M., Braun R.P., Levesque M.P., Dummer R., Goldinger S.M. (2017). Does the Distribution Pattern of Brain Metastases during BRAF Inhibitor Therapy Reflect Phenotype Switching?. Melanoma Res..

[32] Imafuku K., Yoshino K., Yamaguchi K., Tsuboi S., Ohara K., Hata H. (2017). Sudden Onset of Brain Metastasis despite the Use of Vemurafenib for Another Metastatic Lesion in Malignant Melanoma Patients. Case Rep. Oncol..

[33] Ma Y., Wang Q., Dong Q., Zhan L., Zhang J. (2019). How to Differentiate Pseudoprogression from True Progression in Cancer Patients Treated with Immunotherapy. Am. J. Cancer Res..

[34] Walker E.V., Davis F.G. (2019). CBTR founding affiliates Malignant Primary Brain and Other Central Nervous System Tumors Diagnosed in Canada from 2009 to 2013. Neuro-Oncology.

[35] Zakaria D. (2013). An Examination of the NAACCR Method of Assessing Completeness of Case Ascertainment Using the Canadian Cancer Registry. Health Rep..

